# Ununited Fracture Treated by Friction of the Bones

**Published:** 1834

**Authors:** 


12. *Ununited Fracture successfully treated by friction of the ends of the bones upon each other.*—Our experienced and respected friend, Dr. Parish, has published in the journal just quoted, a case of this kind. It was a fracture of the humerus, of several months standing, in which the muscles were greatly relaxed. By pressing the arm firmly, the oblique surfaces were found to slide and grate upon each other. This process was repeated for a number of days, when ossific inflammation was excited, and a union of the bones took place.

